# Marine metagenomics: strategies for the discovery of novel enzymes with biotechnological applications from marine environments

**DOI:** 10.1186/1475-2859-7-27

**Published:** 2008-08-21

**Authors:** Jonathan Kennedy, Julian R Marchesi, Alan DW Dobson

**Affiliations:** 1 Environmental Research Institute, University College Cork, National University of Ireland, Lee Road, Cork, Ireland; 2 Department of Microbiology, University College Cork, National University of Ireland, Western Road Cork, Ireland; 3 School of Biosciences, Cardiff University, Cardiff, UK

## Abstract

Metagenomic based strategies have previously been successfully employed as powerful tools to isolate and identify enzymes with novel biocatalytic activities from the unculturable component of microbial communities from various terrestrial environmental niches. Both sequence based and function based screening approaches have been employed to identify genes encoding novel biocatalytic activities and metabolic pathways from metagenomic libraries. While much of the focus to date has centred on terrestrial based microbial ecosystems, it is clear that the marine environment has enormous microbial biodiversity that remains largely unstudied. Marine microbes are both extremely abundant and diverse; the environments they occupy likewise consist of very diverse niches. As culture-dependent methods have thus far resulted in the isolation of only a tiny percentage of the marine microbiota the application of metagenomic strategies holds great potential to study and exploit the enormous microbial biodiversity which is present within these marine environments.

## Introduction

It has been estimated that pelagic bacteria are extremely abundant, achieving densities of up to 10^6 ^per ml of seawater, and account for most oceanic biomass and metabolism [1]; while numbers of bacteria which are thought to colonize marine snow can reach levels of up to 10^9 ^per ml [2]. Marine environments, including the subsurface are believed to contain a total of approximately 3.67 × 10^30 ^microorganisms [3] and with approximately 71% of the earth's surface of 361 million square kilometers covered by the ocean, this environment represents an enormous pool of potential microbial biodiversity and exploitable biotechnology or "blue biotechnology". This untapped potential has resulted in the recent acceleration in interest in the study of marine microorganisms, with the aim of not only providing us with more information on the key role they play in marine food webs and biogeochemical cycling in marine ecosystems, but also in exploiting their ability to produce novel enzymes and metabolites/compounds with potential biotechnological applications. As with terrestrial environments, where more than 99% of bacteria cannot be cultured by conventional means, the same is true for marine environments where the vast majority of these marine microbes have to date not yet been identified, classified or indeed cultured. According to Amann and colleagues as few as 0.001–0.1% of microbes in seawater are currently cultivable [4]. In this respect the recent advances in culture independent techniques to assess microbial diversity and ecology, such as phylogenetic studies based on small ribosomal RNA (rRNA) analysis and metagenomics, which were initially developed for terrestrial based research are now increasingly being employed in marine environments and are proving extremely useful [5,6]. A clear example of this was the large scale metagenome sequencing project which was recently undertaken on oligotrophic seawater samples from the Sargasso Sea [7] and the Global Ocean Sampling (GOS) expedition [8].

In this review, we highlight the exciting potential that metagenomic based approaches offer us in gaining access to protein-coding genes with biotechnological potential from uncultivable marine microorganisms; thereby allowing us to exploit, to a much greater extent than heretofore, the potential of this vast, and as yet untapped, marine microbial biodiversity resource.

### Metagenomics

**Figure 1:**
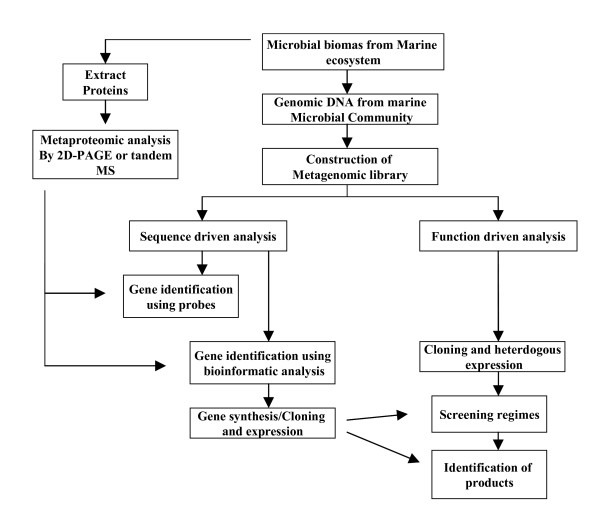
**Omic based approaches to identify novel biocatalysts from marine ecosystems**.

Against the background whereby it is widely believed that more than 99% of bacteria in any given environment cannot be cultured when conventional approaches are employed, metagenomic based approaches have emerged as an attractive option to allow an assessment of the microbial genomes present within these environments [9]. Metagenomics involves the direct cloning of environmental DNA into large clone libraries to facilitate the analysis of the genes and the sequences within these libraries (Figure 1) [10,11]. Metagenomics was initially employed to study non-culturable microbiota and focused primarily on providing a better understanding of global microbial ecology in different environmental niches. With the advent of efficient cloning vectors such as bacterial artificial chromosomes (BACs) and cosmids, together with improved DNA isolation techniques and advanced screening methodologies using robotic instrumentation; it is now possible to express large fragments of DNA and subsequently screen large clone libraries for functional activities [12]. Such approaches have been particularly successful in terrestrial environments, where genes involved in antibiotic production, antibiotic resistance and degradative enzymes have been identified among others [10,13,14]. These approaches coupled with additional innovative screening approaches such as Substrate-Induced Gene Expression screening (SIGEX) have facilitated the cloning of catabolic operons potentially involved in benzoate and catechol degradation among others [15]. These functional based screening approaches have also been supplemented with homology-based screens, primarily involving polymerase chain reaction (PCR)-based approaches targeting novel genes with sequences similar to known genes. This has resulted in the cloning of genes such as polyketide synthases [16], alkane hydroxylases [17], cyclomaltodextrinases [18], xylanases [19] and beta-xylanases [20]. Recently novel methods such as pre-amplification inverse-PCR (PAI-PCR) [21] and metagenomic DNA shuffling [22] have been employed to isolate new biocatalysts. PAI-PCR which has been employed to isolate glycosyl hydrolase genes from horse and termite guts, offers the potential to clone genes for which the copy number of target DNA sequences is low, while the shuffling approach, which has been used to construct novel biocatalysts, simulates and accelerates the evolutionary process using molecular biological tools. Homology-based screening approaches are by definition quite limited, given that homologs of existing genes are being targeted and this often results in no novel gene families being detected. The large scale Global Ocean Sampling Project has revealed that despite the large increase in DNA sequence data we have yet to approach saturation for the discovery of new protein folds, implying that there is a large resource of truly novel proteins and enzymes in uncultured marine microbes [8]. It is thus widely believed that functional-based screening holds far more potential for identifying entirely new enzymes with novel biocatalytic activities [23].

### Marine microbes as good sources of novel biocatalysts

All marine ecosystems are inhabited by microbes, which are both taxonomically diverse and metabolically complex. Marine microbes are both the primary producers of biomass in the oceans, harvesting light and fixing carbon, and the primary recyclers of nutrients. Microbial processes are essential for all the major cycles necessary for the maintenance of ocean life. Marine microbes are also known to be involved in the global cycling of bio-elements such as nitrogen, carbon, oxygen, phosphorous, iron, sulphur and trace elements. The precise contribution of marine microbes to these biogeochemical cycles is unknown. However, because of the versatility of their biochemical capabilities and the vast microbial biomass present in marine ecosystems, they are believed to be the main components responsible for the maintenance of these cycles, which help sustain all living things in these ecosystems. [24].

The marine environment is extremely diverse and marine microbes are exposed to extremes in pressure, temperature, salinity and nutrient availability. These distinct marine environmental niches are likely to possess highly diverse bacterial communities, possessing potentially unique biochemistry. Enzymes isolated from microbes from such environments are likely to have a range of quite diverse biochemical and physiological characteristics that have allowed the microbial communities to adapt and ultimately thrive in these conditions. For example bacteria which colonize marine snow are known to produce extracellular enzymes, whose function is to degrade proteins and polysaccharides within the snow [2]. Thus the potential exists to exploit the enzymes produced by these marine microbes which are likely to possess unique bio-catalytic activities capable of functioning under extreme conditions.

Microbes are also known to form symbiotic relationships with various marine invertebrates within diverse marine ecosystems; including species of sponges, corals, squids, sea squirts and tunicates among others. The symbioses between the marine invertebrates and the microbes are thought to offer each partner a number of advantages. For example, in the case of sponges symbiotic bacteria are believed to provide a source of nutrients for the sponge host, process sponge metabolic waste and produce secondary metabolites which play a role in the overall defence mechanisms of the sponge [25]. These sponges are rich sources of biologically active and pharmacologically valuable natural products, with the potential for therapeutic use, and the bacterial symbionts of these sponges are widely believed to be the producers of many of these products [26,27]. Marine sponges are also known to contain large amounts of halogenated organic compounds such as fatty acids and alkaloids and are thus a potential useful source of both halogenases and dehalogenases a group of biotechnologically important enzymes which can be used in the production of pharmaceuticals, herbicides and pesticides [28-31].

The potential for the discovery of novel enzymes from marine microbes is illustrated by the fact that quite a diverse range of enzymatic activities have to date been identified from cultured marine microbes (Table 1), with the need for novel biocatalysts giving rise to many new enzymes being isolated from the marine environment. These include bacteria isolated from Antarctic seawater and marine sediments among others. Novel enzymes which have recently been identified from marine environments include a non-specific nuclease isolated from a bacteriophage which predates on the marine thermophile *Geobacillus *sp. 6K51. This enzyme has been shown to have no known homology to any previously isolated enzymes and a temperature optimum of 60°C [32]. At the other end of the temperature scale are the cold-adapted enzymes such as the lipase isolated from the γ-proteobacterium, *Pseudoalteromonas haloplanktis *TAC125. This lipase is the first member of a new family of lipases, which share homology to the α/β hydrolases superfamily [33]. Other recently reported enzymes include phospolipases [34], extracelluar amylotic enzymes [35], agarases [36] and endochitinases [37].

**Table 1:** Enzymes from marine microbes

**Enzyme**	**Producing Organism**	**Habitat**	**Ref**
Alanine dehydrogenase	Psychrophilic bacterium strain PA-43	Sea Urchin	[62]
Alcohol dehydrogenase	*Flavobacterium frigidimaris *KUC-1	Antarctic seawater	[63]
Aminopeptidase	*Colwellia psychrorythraea *strain 34H	Marine sediment	[64]
α-amylase	*Nocardiopsis *sp.	Deep sea sediment	[65]
β-Galactosidase	*Arthrobacter *sp. SB	Antarctic sea water	[66]
β-Galactosidase	*Guehomyces pullulans*	Antarctic sea water	[67]
Catalase	*Vibrio salmonicida*	Fish microbiota	[68]
Cellulase	*Pseudoaltermonas haloplanktis*	Antartic sea water	[69]
Cellulase	*Pseudoaltermonas *sp. DY3	Deep-sea sediment	[70]
Chitinase	*Arthrobacter *sp. TAD20	Antarctic ice	[71]
Chitinase	*Rhodothermus marinus*	Marine hot springs	[72]
Esterase	*Vibrio *sp.	Sea Hare eggs	[73]
Epoxide hydrolases	*Erythrobacter litoralis *HTCC2594	Seawater	[74]
Feruloyl esterase	*Pseudoaltermonas haloplanktis*	Antartic sea water	[75]
β-D-glucosidase	*Shewanella *sp. G5	*Munida subrrugosa *(intestine)	[76]
Homoserine transsuccinylase	*Thermotoga maritima*	Marine sediment	[77]
Isocitrate dehydrogenase	*Colwellia psychrerythraea*	Arctic marine sediment	[78]
Isocitrate lyase	*Colwellia psychrerythraea*	Arctic marine sediment	[79]
Lipase	*Pseudoaltermonas haloplanktis *TAC125	Antartic sea water	[33]
Malate dehydrogenase	*Flavobacterium frigidimar *KUC-1	Antartic sea water	[80]
Quinol oxidase	*Shewanella *sp. strain DB-172F	Deep-sea sediment	[81]
Pectate lyase	*Pseudoalteromonas haloplanktis *strain ANT/505	Antarctic sea ice	[82]
Proteases	*Pseudoalteromonas, Shewanella, Colwellia, Planococcus *species	Sub-Antarctic sediment	[83]
Protease (alkaline)	*Pseudomonas strain *DY-A	Deep-sea sediment	[84]
Proteases (serine)	Marine bacterium	Antartic sea water	[54]
Proteases (serine)	*Aeropyrum pernix *K1	Coastal solfataric vent	[85]
Subtilisin	*Bacillus *TA 41	Antartic sea water	[86]
Trehalase	*Rhodothermus marinus*	Marine hot springs	[87]
Uracil-DNA Glycosylase	Marine bacterium strain BMTU3346	Marine sample	[88]
Xylanase	*Pseudoaltermonas haloplanktis*	Antartic sea water	[89]

Even environments such as the deep sea floor, where to date only limited numbers of cultivable bacteria have been isolated, mainly obligate sulphate reducing anaerobes and methanogens together with facultative anaerobic heterotrophs such as *Halomonas *and *Psychrobacter*, now appear to be reservoirs for microbes with enzymatic activities with potential biotechnological applications. For example a number of cultivable aerobic microbes have recently been isolated from the deep subseafloor sediments from off-shore the Shimokita peninsula in Japan at a water depth of 1,180 m. These microbes produced a variety of different enzymatic activities including protease, amylase, lipase, chitinase, deoxyribonuclease and phosphatase activities [38].

However, these novel enzymes have all been isolated from the cultivable fraction of microbes from the diverse range of marine environments which were studied. As we know this cultivable fraction represents only a small proportion of the total bacteria present in these environments. Hence, if the entire potential of these environments is to be explored and ultimately exploited with respect to the presence of novel biocatalysts; then there is a clear need to employ metagenomic or other culture-independent approaches together with robust heterologous expression systems to facilitate such an approach.

### Heterologous expression systems for functional expression of metagenomic libraries from marine environments

The heterologous expression of novel genes or indeed gene clusters within suitable hosts will be required if new biocatalysts are to be identified from unculturable marine sources. However, significant challenges remain in using functional metagenomic based approaches to identify these enzymes; particularly when extreme environments, such as those present in many marine ecosystems, are being targeted. One of the main problems is that of low biomass yields, which coupled with low cell numbers from these marine environments, can lead to difficulties in obtaining high yields of DNA for subsequent cloning. Thus new methods are required to allow metagenomic library construction from environments with only low bacterial cell-densities. One potential method of overcoming this problem is the use of multiple displacement amplification, which has been successfully employed to assess the microbial diversity of scleractinian coral where environmental considerations require minimal sample sizes [39]. In addition, for functional screening to be successful it requires gene expression and proper folding of the resulting protein in a suitable heterologous host. While it has been predicted that *E. coli *expression systems can be employed to successfully express up to 40% of genes from 32 complete genome sequences of various prokaryotic organisms, this is not always easily achievable [40]. For example insolubility of the target protein remains a major limitation in *E. coli *expression systems, while in some structural genomics projects, up to half of the targets tested failed to fold properly and accumulated as insoluble protein or inclusion bodies [41]. With this in mind a number of alternative bacterial host and expression systems are currently being examined. These include *Streptomyces lividans*, *Pseudomonas putida *and *Rhizobium leguminosarum *[42,43], which should facilitate the construction of metagenomic libraries with different expression capabilities thereby overcoming some of the difficulties being encountered with the *E. coli *system. In addition the possibility exists that functional expression of metagenomic DNA from psychrophilic marine microorganisms in heterologous hosts such as *E. coli *may be sub-optimal when the host is cultured at a higher temperature. The expression of proteins from these psychrophilic microorganisms at higher temperatures may lead to the mis-folding of these proteins and the subsequent formation of inclusion bodies, resulting in loss of function. While this may not always be the case [44], in general psychrophilic microorganisms produce enzymes which have become cold-adapted, but which tend to have a low thermal stability. These problems may potentially be overcome through the use of chaperone-based *E. coli *strains bearing the chaperonin 60 gene (cpn60) and the cochaperonin 10 gene (cpn10) from the psychrophilic bacterium *Oleispira antartica *RB8^T ^[45]. The use of these strains for metagenomic library construction could facilitate the functional screening of these libraries at temperatures down to 10°C, where increased levels of expression may in fact be observed [46].

### Successes in the marine environment

Researchers have also begun to employ metagenomic based approaches in an effort to isolate novel compounds from marine environments. Much of the emphasis to date has focused on the identification of gene clusters encoding novel biosynthesis pathways for compounds with potential bio-pharmaceutical applications, from bacterial populations associated with marine invertebrates [25]. The metagenomic approaches being employed are similar to those that have previously been successfully employed in soil, which have resulted in the identification of among others; a biosynthesis gene cluster for the antibiotic violacein [47]; novel *N*-acyltyrosine antibiotics [48]; the novel antibiotic turbomycins [49]; antibiotic compounds related to indirubin [50]; and a family of novel natural products, the terragenines [51]. Successes in the marine environment include identification of the biosynthetic machinery for the cytotoxic peptide patellamide from the cyanobacterial symbiont, *Prochloron*, of a marine didemnid ascidian. Two groups independently identified the biosynthesis genes for patellamide using a DNA sequencing approach and through a functional metagenomics approach. Patellamide biosynthesis was found to proceed though modification of a ribosomally encoded peptide and not via a non-ribosomal peptide synthetases as had been previously assumed [52,53].

With respect to biocatalysts, a number of novel hydrolytic enzymes have recently been cloned from Antarctic sea water bacterial metagenomic DNA [54], while a novel low-temperature-active lipase has also recently been isolated from a metagenomic library of Baltic Sea marine sediment bacteria. This low-temperature-active lipase gene which displayed 54% amino acid similarity to a *Pseudomonas putida *esterase was successfully heterologously expressed in *E. coli *and subsequently biochemically characterised [55]. This highlights the value of employing metagenomics in marine environments to, in this case, identify novel lipases. Lipases have important applications not only in the detergent industry but also in paper processing, as food additives [56] and in biofuel production through catalysing the conversion of vegetable oil to methylalcohol ester [57]. They also have applications in synthetic organic chemistry, due primarily to their enantio-/stereoselectivity coupled with their ability to retain activity in organic solvents. Thus similar metagenomics approaches in other marine environments may prove fruitful in identifying other novel lipase genes, with biotechnological applications in these areas.

Another example where metagenomics has been successfully employed to identify novel enzymes from a marine environment is the recent report of the cloning of two alkane hydroxylase genes from a metagenomic library from deep-sea sediment in the Pacific. While this is the first report of the genetic characterisation of an alkane hydroxylase from a deep-sea environment, it is also interesting to note that these two alkane hydroxylase genes were functionally expressed in a *Pseudomonas fluorescens *strain [17]. Identification of these novel proteins may help to increase the range of alkane hydroxylase biocatalytic applications, and again highlights the utility of metagenomics in identifying potential novel biocatalysts.

### Challenges and future directions

There are several key areas which need to be addressed in order for this exciting area to realise its full potential. The high microbial diversity of these marine environments means that large numbers of clones need to be screened in order to access the full biodiversity of the microbial community and to obtain significant numbers of "hits" which can then be taken forward for further analysis. This requirement means that high throughput screens need to be established to identify positive clones in metagenomic libraries. The development of cell-based ultra-high throughput screens would enable more rapid screening of large metagenomic libraries. Flow cytometry based approaches such as the SIGEX approach are leading the way in this area, since millions of clones can be screened in a short time and sorted for further analysis.

While standard metagenomic approaches allow access to the full biodiversity of microbial populations, the nature of the metagenomic approach means that to access low abundance (< 1%) microbes, extremely large libraries are required. These large libraries contain significant redundancy with respect to the high abundance microbes present within the population. A combination of cell sorting technology (FACS and microfluidics) and whole genome amplification approaches has the potential to significantly improve the ability to access the genetic resources of low-abundance microbes within any given population [58-60]. These new approaches to access low abundance microbial diversity could be used to make targeted 'metagenomic' libraries of low abundance microbes and increase our ability to access this diversity.

The functional metagenomic approach, typically used for biocatalyst screening, requires that the desired activity be expressed in a surrogate host, typically *Escherichia coli*. While *E. coli *has proven to be a flexible and useful host for heterologous expression there are a significant proportion of proteins that cannot be expression functionally in this host. In addition, many functional assays rely on the expression of entire metabolic pathways, requiring that promoters be recognised in the heterologous host for the co-ordinated expression of entire sets of genes. The range of surrogate hosts for heterologous expression and their associated vectors needs to be greatly expanded if we are to succeed in expressing DNA from the many diverse phyla that exist in the marine ecosystem, including the abundant Archaea and microeukarya. Novel complementation assays need to be developed whereby the metagenomic DNA complements a mutation in a surrogate host. Such methods would be very high throughput since they would allow the positive selection of desired clones. The ability to screen extremely large libraries also makes the use of easier to construct small insert libraries more practical.

While metagenomic approaches offer a means to access the total potential of the microbial gene pool, other approaches need to be considered which may offer new avenues of investigation. One such approach may be to explore the metaproteome of the marine microbes (Fig 1). In metaproteomics the proteins present in an environmental sample are analysed using high throughput methods such as tandem mass spectroscopy [61], comparison of this data to metagenomic DNA sequence data allows one to infer which genes are actively expressed in any population. Using appropriately designed probes this approach would allow the selection of such active genes for the screening and analysis of metagenomic libraries. Using such an approach in tandem perhaps with the more established metagenomic based approaches would greatly enhance the probability of obtaining novel biocatalysts.

## Conclusion

This review is intended to focus the reader's attention on the potential of exploiting metagenomics, specifically the use of metagenomic libraries constructed from unique marine environments as an approach to successfully exploit the largely "untapped" resources within various marine environments. The marine environment is extremely diverse with microbes flourishing in cold polar and warm equatorial waters, in sunlit surface waters, in high-pressure deep sea sediments, in hot acidic water near hydrothermal vents and in association with various vertebrate and invertebrate animals. These diverse ecosystems are potentially very useful sources for novel enzymes with unique properties and great biotechnological potential. Only a small proportion of the bioresources of marine microbiota have thus far been examined and an even smaller proportion has been exploited. Given our present inability to culture the vast majority of microbes from these environments the metagenomic approaches outlined in this review offer the only methodology currently available to access these unique and useful bioresources. There is an ongoing need for a wide range of novel biocatalysts which are required to improve current and develop new, cleaner, industrial production processes, to reduce energy and raw material consumption, and for the generation of renewable biofuels; marine metagenomics coupled with biotechnology has the potential to contribute to all these pressing needs.

## Competing interests

The authors declare that they have no competing interests.

## Authors' contributions

ADWD drafted the manuscript. JK and JRM contributed additional content throughout the article. All authors have read and approved the final manuscript.
